# Biomedical Vocabulary Alignment at Scale in the UMLS Metathesaurus

**DOI:** 10.1145/3442381.3450128

**Published:** 2021-04-19

**Authors:** Vinh Nguyen, Hong Yung Yip, Olivier Bodenreider

**Affiliations:** National Library of Medicine, Bethesda, Maryland, USA; University of South Carolina, Columbia, South Carolina, USA; National Library of Medicine, Bethesda, Maryland, USA

**Keywords:** UMLS Metathesaurus, neural networks, vocabulary alignment, scalability, supervised learning, logical rules

## Abstract

With 214 source vocabularies, the construction and maintenance process of the UMLS (Unified Medical Language System) Metathesaurus terminology integration system is costly, time-consuming, and error-prone as it primarily relies on (1) lexical and semantic processing for suggesting groupings of synonymous terms, and (2) the expertise of UMLS editors for curating these synonymy predictions. This paper aims to improve the UMLS Metathesaurus construction process by developing a novel supervised learning approach for improving the task of suggesting synonymous pairs that can scale to the size and diversity of the UMLS source vocabularies. We evaluate this deep learning (DL) approach against a rule-based approach (RBA) that approximates the current UMLS Metathesaurus construction process. The key to the generalizability of our approach is the use of various degrees of lexical similarity in negative pairs during the training process.

Our initial experiments demonstrate the strong performance across multiple datasets of our DL approach in terms of recall (91-92%), precision (88-99%), and F1 score (89-95%). Our DL approach largely outperforms the RBA method in recall (+23%), precision (+2.4%), and F1 score (+14.1%). This novel approach has great potential for improving the UMLS Metathesaurus construction process by providing better synonymy suggestions to the UMLS editors.

## 1 INTRODUCTION

### Motivation.

Developed by the National Library of Medicine, the UMLS (Unified Medical Language System) Metathesaurus [4] is a terminology integration system constructed by integrating biomedical terms from over 200 source vocabularies and organizing them into concepts consisting of clusters of synonymous terms from the source vocabularies. The basic building block of the Metathesaurus, also known as an “atom,” is a term from a source vocabulary.

In practice, synonymous atoms are assigned the same concept unique identifier (CUI). Such concepts can be thought of as equivalent mappings from an ontology alignment perspective. In fact, a subset of three source vocabularies from the Metathesaurus (NCI, FMA, and SNOMED CT) have been used by the Ontology Alignment Evaluation Initiative (OAEI) since 2011 [2] and in related efforts [18, 21]. The OAEI aims to compare ontology matching systems on defined test cases. The OAEI organizers have used UMLS synonymy information from the Metathesaurus concepts as reference mappings for biomedical ontologies integrated in the UMLS. Although the Metathesaurus construction process may have similarities to ontology alignment, not all source vocabularies in the Metathesaurus are well-defined ontologies formally represented in OWL. Therefore, in order to avoid any misunderstanding especially in the context of the Semantic Web, we will continue to use the term *vocabulary* instead of ontology when referring to source vocabularies in the Metathesaurus.

The Metathesaurus construction process is based on the assumption that specially trained human experts can determine synonymy among atoms with high accuracy from the candidates obtained from a lexical similarity model and semantic pre-processing. However, manual curation is error-prone as pointed out by [7, 8, 19, 30, 31]. Given the current size of the Metathesaurus with 15.5 million atoms from 214 source vocabularies grouped into 4.28 million concepts, its maintenance process is costly, time-consuming, and extremely demanding on the human expert editors. On the other hand, with the enormous knowledge accumulated over 30 years of manual curation, the existing Metathesaurus provides ample material for supervised learning.

Supervised learning approaches with word embeddings have shown promising results in previous Metathesaurus-related experiments confirming that they have reasonably good performance for the alignment of a selected subset of source vocabularies in the Metathesaurus [21, 45, 47, 48]. In this work, we propose to use these techniques to predict synonymy from all source vocabularies in the Metathesaurus. Aligning over 214 vocabularies with their large size and vast diversity introduces new challenges compared to the OAEI task of aligning a few vocabularies.

In this work, we are mostly interested in assessing the feasibility of using deep learning (DL) techniques for terminology integration at scale in the UMLS Metathesaurus. Therefore, this investigation is not primarily technical and does not have the usual features of a DL benchmarking study. Instead, we investigate whether a simple DL approach can outperform the editorial rules established for building the UMLS Metathesaurus.

### Objectives.

Our primary objective is to develop a scalable supervised learning approach to improve synonymy predictions compared to the current lexical and semantic processing in the Metathesaurus. While existing ontology alignment approaches [2, 18, 21, 47] have been successful on small subsets of 3 to 8 source vocabularies, our goal is to develop an approach that scales not only to large numbers of source vocabularies, but also to diverse source vocabularies, such as those in the Metathesaurus. We expect such a supervised learning approach to outperform a rule-based approach (RBA) that approximates the lexical and semantic processing used in the current Metathesaurus construction process. We will explain the rule-based approximation in Section 3.2.

Our secondary objective is to investigate the extent to which lexical similarity between the atoms used for training influences the performance of our algorithm. Intuitively, it seems more difficult to predict the absence of synonymy between lexically-similar atoms than between lexically-different atoms. We hypothesize that learning from pairs with different degrees of lexical similarity will help improve the performance and generalization of the algorithm.

### Contribution.

Our contributions include:

- The first attempt to define and address terminology integration at the full scale and diversity of the UMLS Metathesaurus using a learning-based approach.
- A reusable rule-based baseline approximating the current lexical and semantic processing used in the UMLS for comparing the performance of our algorithm against the current UMLS building process.
- A generalizable supervised learning approach that is shown to largely outperform the current lexical and semantic processing used in the UMLS Metathesaurus construction process.
- A confirmed hypothesis that the variety of degrees of lexical similarity in negative pairs from the training set is the key to the generalizability of the algorithm.

The remainder of the paper is organized as follows. Section 2 provides relevant background knowledge about the Metathesaurus. Section 3 describes the synonymy prediction and the rule-based approximation as a proxy to the current Metathesaurus construction process. Section 4 describes our supervised learning approach. In section 5, we present our experiments and discuss their results. In section 6, we discuss related work. Section 7 concludes the paper.

## 2 BACKGROUND: KNOWLEDGE REPRESENTATION IN THE UMLS METATHESAURUS

This section presents background knowledge about the UMLS Metathesaurus [4] necessary for describing and understanding the synonymy prediction task, as well as the rule-based approximation of the Metathesaurus building process. We will use the examples in Table 1 to illustrate the concept structure in the Metathesaurus.

As mentioned earlier, key to the UMLS Metathesaurus are the notions of atom (a term from a specific source vocabulary, identified with a specific source concept identifier) and concept (grouping of synonymous atoms). While the Metathesaurus preserves source concept identifiers (SCUI), it also assigns its own identifiers to atoms (AUI), unique strings (SUI), normalized strings (LUI) and concepts (CUI). Table 1 shows examples of atoms and the various types of identifiers they were assigned. Additionally the Metathesaurus editors assign semantic types to each UMLS concept to denote the broad semantics of each concept. Of note, semantic types are not assigned to AUIs, but to CUIs instead. However, it is possible to approximate the semantics of an atom by inferring it from that of the source vocabulary (for semantically homogeneous vocabularies, such as anatomy ontologies), or the top-level subdivisions of a vocabulary (for broad-coverage vocabularies).

Let us consider three tuple pairs (*t*_1_, *t*_3_), (*t*_4_, *t*_5_), and (*t*_1_, *t*_5_) from Table 1 with

*t*_1_ = (“Headache”, “MSH”, “M0009824”, “Disorders”)
*t*_3_ = (“Cranial Pains”, “MSH”, “M0009824”, “Disorders”)
*t*_4_ = (“Cephalodynia”, “MSH”, “M0009824”, “Disorders”)
*t*_5_ = (“Cephalodynia”, “SNOMEDCT_US”, “25064002”, “Disorders”).

In the UMLS Metathesaurus, the information available to the construction process are input tuples in the form of (*str, src, scui, sg*) where *str* is the original string from the source *src*, and *scui* is the optional identifier of that *str* string from the source *src*, and *sg* is a semantic group reflecting the semantics of the string in the source. Of note, for this experiment, we manually assigned one semantic group to each vocabulary and to the top-level subdivisions of heterogeneous vocabularies. Each atom inherits its semantic from its source or from its high-level ancestor(s).

Let *T* = (*S_STR_, S_SRC_, S_SCUI_, S_SG_*) be the set of all input tuples in the Metathesaurus where *S_STR_* is the set of all strings, *S_SRC_* is the set of all sources, *S_SCUI_* is the set of all source concept unique identifiers, and *S_SG_* is the set of all semantic groups. The tuples *t*_1_ = (“Headache”, “MSH”, “M0009824”, “Disorders”) and *t*_3_ = (“Cranial Pains”, “MSH”, “M0009824”, “Disorders”) are instances of *T*. Given the input tuple pairs (*sfr, src, scui, sg*) and (*str′, src′, scui′, sg′*) as instances of *T* = (*S_STR_, S_SRC_, S_SCUI_, S_SG_*) from source vocabularies, the Metathesaurus defines several identifier types for characterizing atoms during the integration process.

### AUI and *m_a_* link mapping function.

The basic building blocks or “atoms” from which the Metathesaurus is constructed are the concept names or strings from each of the source vocabularies. Every occurrence of a string in each source vocabulary is assigned a unique atom identifier (AUI). When the same string appears in multiple source vocabularies, for example, “Cephalodynia” appearing in both MSH and SNOMEDCT_US, they are assigned different AUIs “A26628141” and “A2957278” as shown in Table 1.

(D1) Let *S_AUI_* be the set of all AUIs in the Metathesaurus. Let *m_a_* be the function that maps concept string *str* ∈ *S_STR_* from source vocabulary *src* ∈ *S_SRC_* to a new AUI *a* ∈ *S_AUI_* such that *a* = *m_a_* (*str, src*).

### SUI and *m_s_*.

These AUIs are then linked to a unique string identifier (SUI) to represent occurrences of the same string. Any lexical variation in character set, upper-lower case, or punctuation will result in a separate SUI. For example, the strings “Headache” and “Headaches” are linked to two different SUIs.

(D2) Let *S_SUI_* be the set of all SUIs in the Metathesaurus. Let *m_s_* be the function that maps an AUI *a* ∈ *S_AUI_* to a new SUI *s* ∈ *S_SUI_* such that *s* = *m_s_*(*a*).

### LUI and *m_l_*.

All the English lexical variants of a given string (detected using the Lexical Variant Generator tool [26]) are associated with a single normalized term (LUI). The LVG tool recognizes that the two strings “Headache” and “Headaches” only differ by minor lexical variation and associates them with the same LUI “L0018681”.

(D3) Let *S_LUI_* be the set of all LUIs in the Metathesaurus. Let *m_l_* be the function that maps a SUI *s* ∈ *S_SUI_* to a new LUI *l* ∈ *S_LUI_* such that *l* = *m_l_*(*s*).

### CUI.

Lexical similarity forms the basis for suggesting synonymy in the UMLS Metathesaurus. However, all atoms that share the same LUI are not necessarily synonymous. For example, the string “nail” can denote both an anatomical structure and a surgical device. Table 1 illustrates how synonymous terms are clustered into the same concept (CUI = “C0018681”). Note that we do not define the link mapping from AUI to CUI here because this link is unavailable to the task and cannot be used in the prediction function.

### SCUI and *m_u_*.

Each AUI is optionally associated with one identifier providedby its source (SCUI). Several strings including “Headache”, “Headaches”, “Cranial Pains”, and “Cephalodynia” are associated with the same SCUI, “M0009824”, from the source vocabulary MSH. SCUIs play an important role in the Metathesaurus construction process because source synonymy is very often conserved in the Metathesaurus.

(D4) Let *S_SCUI_* be the set of all SCUIs in the Metathesaurus. Let *m_u_* be the function that maps a concept string *a* ∈ *S_AUI_* to a new SCUI *u* ∈ *S_SCUI_* such that *u* = *m_u_*(*a*).

### Semantic Group and *m_g_*.

As mentioned earlier, semantic groups (or semantic types) are assigned to CUIs, not AUIs, by the Metathesaurus editors. For this reason, this information is unavailable to the task and cannot be used in the prediction function. Instead, we manually assigned semantic groups to source vocabularies or to their top-level subdivisions. All the atoms from a source vocabulary (or top-level subdivision thereof) inherit the semantic group of the source (or top-level subdivision). Most of the atoms have a single semantic group. Semantic group information is used to determine semantic compatibility among atoms defined as sharing one semantic group.

(D5) Let *S_SG_* be the set of all semantic groups in the Metathesaurus. Let *m_g_* be the function that maps concept string *a* ∈ *S_AUI_* to a set of semantic groups *g* ⊂ *S_SG_* such that *g* = *m_g_*(*a*).

So far we have defined the constraint mappings for each AUI to be linked to other identifier types. Every AUI is linked to a single string STR, a single SCUI (optionally), a single SUI, a single LUI, and, most often, a single Semantic Group. Next we will show how these identifiers and mapping links can be leveraged in the rule-based approximation of the Metathesaurus construction process to derive synonymy predictions.

## 3 PROBLEM FORMULATION AND RULE-BASED APPROXIMATION BASELINE

### 3.1 Problem Formulation

We define the synonymy prediction task as follows. *T* is the set of all input tuples (*S_STR_, S_SRC_, S_SCUI_, S_SG_*) from source vocabularies.

Let *t* = (*str, src, scui, sg*) ∈ *T*, and *t′* = (*str′, src′, scui′, sg′*) ∈ *T*. Let *p*: *T* × *T* → {0,1} be the prediction function mapping a pair of input tuples to either 0 or 1. The two strings *str* from *t* and *str′* from *t′* are synonymous if *p*(*t,t′*) = 1.

Note that here we consider the whole tuple for the prediction task instead of using the string *str* only. A string itself does not carry sufficient information for the task at hand; we need to know which source the string comes from and which semantics it has. This is especially useful for processing homonyms (e.g., depending on the source, “nail” can denote an anatomical structure or a surgical device, which will be indicated by the semantic group, “Anatomy” or “Device”).

As ground truth for the prediction task, we use the groupings of strings into concepts in the Metathesaurus. If two strings from two different tuples are assigned the same CUI, they are synonymous. Otherwise, they are not.

A synonymy prediction task will decide if each of the tuple pairs is synonymous (or, more precisely, if the atoms in each pair are synonymous). Finding the prediction function *p* is the problem we address in this paper. We will describe the rule-based approach in Section 3.2 and the supervised learning approach in Section 4.

### 3.2 Rule-based Approximation of the Metathesaurus Construction Process

Here we formalize an approach that approximates the current Metathesaurus construction process that takes as input tuple pairs from source vocabularies. (We have confirmed with the UMLS Metathesaurus editors at the National Library of Medicine that this formalization of the Metathesaurus editorial guidelines accurately reflects the Metathesaurus construction process.) We use this approximation as a baseline in the evaluation of our supervised learning approach. We use the concepts/identifiers and functions/links described in Section 2 to show how the identifiers and links can be combined into rules for synonymy predictions.

We have defined *S_STR_, S_SRC_, S_SCUI_, S_AUI_, S_SUI_, S_LUI_, S_CUI_*, and *S_SG_* to be the set of all strings, sources, SCUIs, AUIs, SUIs, LUIs, CUIs, and semantic groups in the Metathesaurus, respectively. We also have the link mapping functions *m_a_, m_s_, m_l_, m_u_*, and *m_g_* defined from (D1), (D2), (D3), (D4), and (D5) above. Next we will derive the editorial rules from the identifiers and mapping links in the Metathesaurus.

The rule-based approach reflects the following Metathesaurus construction principles:

- Synonymy asserted between atoms in a source vocabulary tends to be conserved in the Metathesaurus
- Lexical similarity is used to identify candidates for synonymy
- Atoms that do not share a common semantics are prevented from being recognized as synonymous and grouped into the same concept

These principles are formalized into two rules, “source synonymy” and “lexical similarity and semantic compatibility”. These rules can be combined into a disjunction and amplified through transitivity.

To illustrate the rule-based approach, we will evaluate the tuple pairs (*t*_1_, *t*_3_), (*t*_4_, *t*_5_), and (*t*_1_, *t*_5_) from Table 1 against each rule with

*t*_1_ = (“Headache”, “MSH”, “M0009824”, “Disorders”),
*t*_3_ = (“Cranial Pains”, “MSH”, “M0009824”, “Disorders”),
*t*_4_ = (“Cephalodynia”, “MSH”, “M0009824”, “Disorders”),
*t*_5_ = (“Cephalodynia”, “SNOMEDCT_US”, “25064002”, “Disorders”).

#### Source synonymy (SS) rule.

The two input tuples are synonymous if they have the same identifier in a given source (SCUI). Formally, given a tuple pair *t* = (*sfr, src, scui, sg*) ∈ *T* and *t*′ = (*str′, src′, scui′, sg′*) ∈ *T*, let *p_ss_* be the prediction function for the source synonymy rule: if *scui* = *scui′* then *p_ss_* (*t, t′*) = 1.

For the example at hand, *p_ss_* (*t_1_*, *t*_3_) = 1, *p_ss_* (*t*_4_, *t*_5_) = 0, *p_ss_* (*t*_1_, *t*_5_) = 0. *t*_1_ and *t*_3_ are predicted to be synonymous because they share the same SCUI “M0009824” from MSH.

#### Lexical similarity and semantic compatibility (LS_SC) rule.

The two input tuples are synonymous if they have the same lexical terms and semantic groups derived from the input tuples using the set of identifiers and links in the Metathesaurus. In practice, given the input as a pair of tuples, included in the lexical similarity and semantic compatibility rule are: (1) a set of axioms to derive the lexical term (*lui, lui′*) and semantic groups (*sg, sg′*) for each input tuple, and (2) the assertions that they have the same lexical term and a common semantic group. We formalize this rule using the Metathesaurus notions as follows.

Given a tuple pair *t* = (*str, src, scui, sg*) ∈ *T* and *t′* = (*str′, src′, scui′, sg′*) ∈ *T*, let *p_lssc_* be the prediction function for the lexical and semantic similarity rule: *p_lssc_*(*t,t′*) = 1 if

(1) *aui* = *m_a_*(*str, src*), *sui* = *m_s_*(*aui*), *lui* = *m_l_*(*sui*), *sg* = *m_g_*(*aui*), (deriving lui and sg)

*aui*′ = *m_a_* (*str′, src′*), *sui′* = *m_s_* (*aui′*), *lui′* = *m_l_*(*sui′*), *sg′* = *m_g_* (*aui′*),

(2) *lui* = *lui′* and *sg* ∩ *sg′* ≠ ∅ (asserting lui and sg).

For the current example, *p_lssc_*(*t*_1_, *t*_3_) = 0, *p_lssc_* (*t*_4_, *t*_5_) = 1, *p_lssc_* (*t*_1_, *t*_5_) = 0, *t*_4_ and *t*_5_ are predicted to be synonymous because they share the same LUI “L0380797” and semantic group “Disorders”.

#### Rule combination (SS_LS_SC).

For the three tuple pairs at hand, the two pairs (*t*_1_, *t*_3_) and (*t*_4_, *t*_5_) are predicted to be synonymous by the source synonymy rule and the lexical and semantic similarity rule. The last pair (*t*_1_, *t*_5_) is predicted to be non-synonymous by both rules. However, all these pairs share the same CUI and are considered synonymous in the Metathesaurus (ground truth). Therefore, the rule-based approach can only correctly predict two out of the three pairs above.

Since both source synonymy preservation and lexical and semantic similarity are principles used in the Metathesaurus construction process, it is legitimate to create a disjunction of the corresponding rules (i.e., SS or LS_SC).

Given a tuple pair *t* = (*str, src, scui, sg*) ∈ *T* and *t′* = (*str′, src′, scui′, sg′*) ∈ *T*, let *p_sslssc_* be the prediction function for the source synonymy and the lexical and semantic similarity rule: *p_sslssc_* (*t, t′*) = 1 if *p_ss*(*t, t′*) = 1 or *p_lssc*(*t, t′*) = 1.

#### Transitivity.

The combination rule *SS_LS_SC* can be further amplified by considering its transitive closure. Given *t*_1_, *t*_2_, *t*_3_ ∈ *T*, let *p_trans_* be the prediction function for the transitivity rule: *P* = {*p_ss_, p_lssc_, p_sslssc_, p_trans_*} is the set of prediction functions, *p_trans_* (*t*_1_, *t*_3_) = 1 if ∃ *p*_1_, *p*_2_ ∈ *P* such that *p*_1_ (*t*_1_, *t*_2_) = 1 and *p_2_* (*t*_2_, *t*_3_) = 1.

Note that all prediction functions in *P* are commutative. Changing the order of the parameters does not change the results. Section 5 will describe our experiments and evaluate this approach against the supervised learning approach described in Section 4.

## 4 SUPERVISED LEARNING APPROACH

This section introduces our supervised approach for learning and predicting synonymy among Metathesaurus atoms. The general idea is to learn similarities between pairs of atoms within a concept and dissimilarities between pairs of atoms across concepts. We present the model formulation, dataset generation and neural network architecture. Table 2 provides a list of abbreviations used in the paper for a quick reference.

### 4.1 Problem Formulation

Supervised deep learning (DL) is a learning function that maps an input to an output based on examples of input-output pairs through layers of dense networks [39]. The Metathesaurus comprises approximately 10 million English atoms, each of which is associated with a concept. One can simply train a supervised classifier to predict which concept should be assigned to a given atom. However, this approach is considered an extreme classification task [3] due to the very large prediction space of 4.28 million concepts. However, the concept is simply a “mechanism” to cluster synonymous atoms together. We are primarily interested in assessing whether two atoms are synonymous and should be labeled with the same concept regardless of whether this concept already exists in the Metathesaurus. Hence, we formulate this problem as a similarity task. Ideally, we would like to to assess similarity based not only on the lexical features of an atom, but also on its context (e.g., represented by neighboring concepts in this source vocabulary). However, in this preliminary investigation, we only rely on the term itself to determine synonymy among atoms. In practice, a fully-trained model should identify and learn scenarios where

- Atoms that are **lexically similar** in nature but are **not synonymous**, e.g., “Lung disease and disorder” versus “Head disease and disorder”, and
- Atoms that are **lexically dissimilar** but are **synonymous**, e.g., “Addison’s disease” versus “Primary adrenal deficiency”.

Moreover, such a model should outperform the current Metathesaurus building process, approximated by the rule-based approach described earlier.

### 4.2 Dataset generation

The input data for supervised learning is the same as for the rule-based approach, with the difference that supervised learning only relies on the terms, while the rule-base approach also uses some elements of context (source synonymy and semantic group). In both cases, we use the active subset of the 2020AA UMLS. Only atoms from English source vocabularies are used, excluding atoms marked as suppressible synonyms. The final dataset consists of 8.7M strings from 168 sources grouped into 4.2M concepts.

#### Ground truth.

Labeled data are taken from the pairs of atoms that are linked to the same (positive) or different (negative) concepts. Let *POS* be the set of positive pairs and *NEG* be the set of negative pairs. Given a pair of tuples *t* = (*str, src, scui, sg*) and *t′* = (*str′, src′, scui′, sg′*), *aui* = *m_a_*(*str, src*), *aui′* = *m_a_* (*str′, src′*), let *m_c_* be the mapping function respectively linking *aui, aui′* ∈ *S_AUI_* to *cui, cui′* ∈ *S_CUI_* such that *cui* = *m_c_* (*aui*) and *cui′* = *m_c_* (*aui′*), if *cui* = *cui′* then (*aui, aui′*) ∈ *POS* else (*aui, aui′*) ∈ *NEG*.

The number of positive pairs in *POS* is approximately 27.9M, and the number of negative pairs in *NEG* is approximately 10^14^ since most atoms do not share a CUI. It is computationally impossible for us to generate all of the negative pairs in *NEG.* Even if we could overcome resource limitations, training with extreme class imbalance towards negative is unlikely to yield accurate predictions. Therefore, we drastically reduce the negative sample space so that the datasets have a better class balance.

#### Data generation principles.

We follow two principles to generate the experimental datasets: (1) provide different degrees of lexical similarity in the negative pairs, and (2) maximize the coverage of AUIs in the training datasets.

We hypothesize that neural networks can predict more efficiently if they can learn from interesting negative pairs that are lexically similar. However, since most negative pairs have no (or low) lexical similarity, it is particularly important for the algorithm to learn from lexically-similar negative pairs. Therefore, we created various negative sets with different levels of lexical similarity so that we can assess how lexical similarity influences performance.

We also hypothesize that neural networks can generalize better if they can learn from both positive and negative pairs for every string in the Metathesaurus. We would also like to maintain the class balance (i.e., keep the maximum ratio between positive and negative pairs at about 1:3). Therefore, every atom in the Metathesaurus will have *n* positive pairs and approximately ≤ 3*n* negative pairs.

We use the Jaccard index (1) as a measure for the similarity between atoms. To ignore minor variation among atoms (e.g., singular/plural differences), we assess the lexical similarity of normalized strings rather than original strings. Let *norm* be the normalizing function that maps a *sui* to its normalized string, and *m_s_* be the function mapping an AUI to its SUI. The JACC score assessing the similarity between two AUIs is computed as follows.

(1) $$JACC(aui,aui^{\prime })=\frac{\left|norm(m_{s}(aui))\cap norm(m_{s}(aui^{\prime }))\right|}{\left|norm(m_{s}(aui))\cup norm(m_{s}(aui^{\prime }))\right|}$$

For example, using normalized words from atoms from Table 1, JACC(“A0066000”, “A0066008”) = 1/1 = 1.0 (1 word total; 1 word in common). JACC(“A0066000”, “A1641924”) = 0/3 = 0 (3 words total; no words in common). JACC(“A0066000”, “A3487586”) = 1/3 = 0.33 (3 words total; 1 word in common).

#### Degrees of similarity in negative pairs.

We can divide all of the negative pairs in the Metathesaurus into two mutually exclusive sets: (1) SIM, the negative pairs with some similarity (JACC > 0) between the two atoms, and (2) NOSIM, the negative pairs that have no similarity (JACC = 0) between the two atoms. We can formally define these sets as follows.

Given a pair of tuples *t* = (*str, src, scui, sg*) and *t′* = (*str′, src′, scui′, sg′*), with *aui* = *m_a_*(*str, src*), *aui′* = *m_a_*(*str′, src′*), and (*str, str′*) ∈ *NEG*, if *JACC(aui, aui′*) > 0, then (*aui, aui′*) ∈ SIM, else (*aui, aui′*) ∈ NOSIM.

In practice, the size of the SIM set is significantly smaller than that of the NOSIM set.

#### Variants of the negative dataset.

Using the two principles described above, we create four variants of the negative dataset as follows. *NEG_TOPN_*(*SIM*): negative pairs with the highest similarity scores. *NEG_RAN_*(*SIM*): random negative pairs having some similarity. *NEG_RAN_*(*NOSIM*): random negative pairs having no similarity. *NEG_ALL_* = *NEG_TOPN_*(*SIM*) ∪ *NEG_RAN_*(*SIM*) ∪ *NEG_RAN_*(*NOSIM*): include all of the above pairs.

Formally, the number of positive and negative pairs in each dataset variant is computed as follows. Let *m_c_* be the ground truth function mapping an AUI *a* to its concept CUI *c, c* = *m_c_* (*a*). Let *m_ca_* be the function mapping a CUI *c* to its AUIs *a*, then *m_ca_*(*c*) = {*a* : *c* = *m_c_* (*a*)}. Let *n* be the number of AUIs within a CUI, then *n*(*a*) = |*m_ca_*(*m_c_* (*a*))| = |{*a′* : *c* = *m_c_*(*a′*)}|. Let (*a, a′*) be an ordered pair of AUIs, then for every AUI *a* having *k* = (*n*(*a*) – 1) positive pairs (a,*). *NEG_TOPN_* (*SIM*) includes 2*k negative pairs (a,*) or only 1 negative pair if k = 0. *NEG_RAN_*(*SIM*) includes 2*k negative pairs (a,*) or only 1 negative pair if k = 0. *NEG_RAN_* (*NOSIM*) includes 2*k negative pairs (a,*). *NEG_ALL_* includes up to 6*k negative pairs (a,*).

If there is a single atom in a concept, no positive pairs can be created (k=0). In such cases, we will add a negative pair for this atom to *NEG_TOPN_*(*SIM*) and *NEG_RAN_* (*SIM*) if this atom shares at least some similarity with other atoms. Note that we select twice as many negative pairs as needed for training purposes in each set so that we can split each set of negative pairs equally between learning and generalization experiments.

#### Learning vs. generalization datasets.

We create two types of datasets: (1) learning datasets for training and validating the neural network models, and (2) generalization datasets for testing the generalization of the neural network models. The datasets of the two types are mutually exclusive.

In summary, as shown in Table 3, we create 4 dataset variants (TOPN_SIM, RAN_SIM, RAN_NOSIM, and ALL) for each dataset type. We split the set of positive pairs, POS, randomly into the learning and generalization datasets (80:20 ratio). The positive learning datasets (80% of POS) will be combined with the one half of the negative dataset for a given variant. Similarly, the positive generalization datasets (20% of POS) will be combined with other half of the negative datasets for a given variant. Therefore, the size of the learning datasets are bigger than the generalization datasets because they have more positive pairs. Hence, we have 8 datasets in total as shown in Table 3 for the experiments in Section 5.

### 4.3 Neural Network Architecture

**Figure 1:**
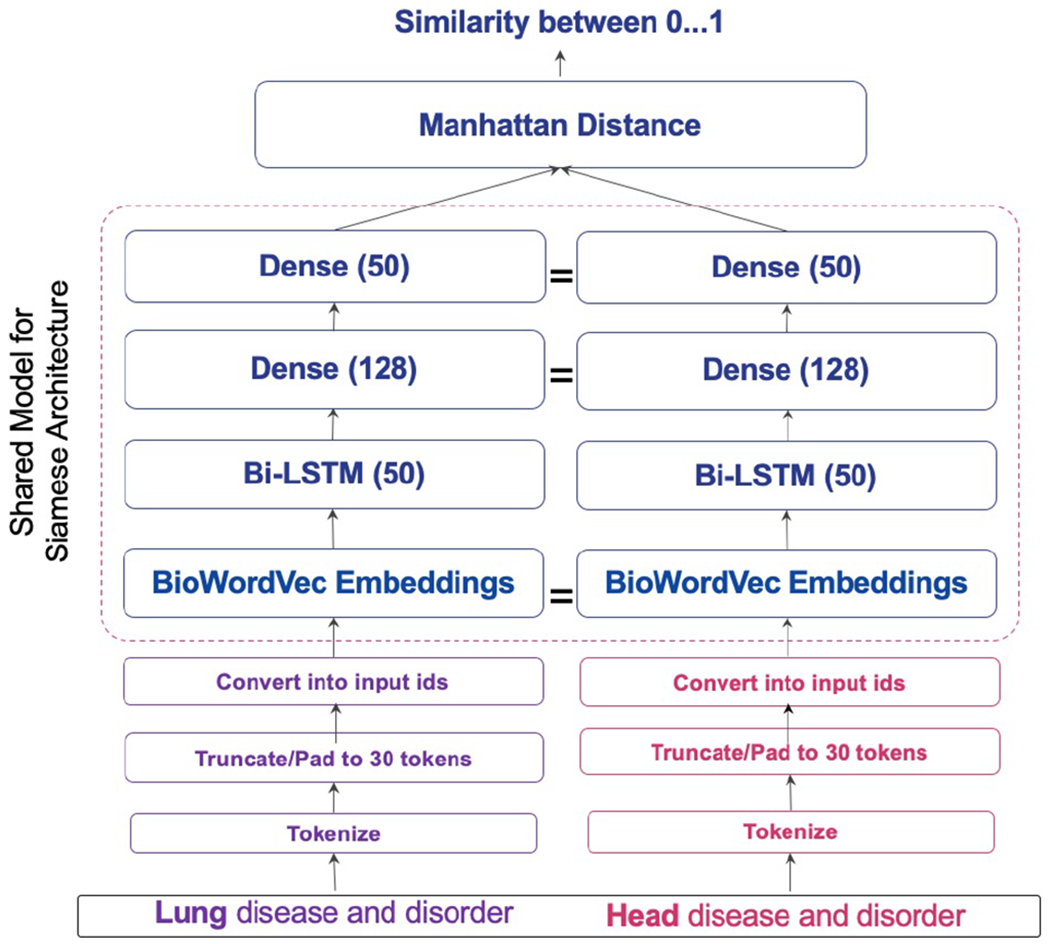
Neural network architecture with Siamese network and BioWordVec embeddings.

Our model adopts the Siamese structure from [32] with BioWordVec embeddings as shown in Figure 1.

#### Word embeddings.

A pair of atoms are first transformed into their respective numerical word representations, i.e., word vectors. A word embedding is a language modeling and feature learning technique in NLP where words are mapped to vectors of real numbers with varying dimensions. These word vectors are positioned in the vector space such that words that share similar contexts in the corpus are situated close to one another in the space [28]. Word embeddings are often used to calculate sentence pair similarity. In the general domain, the SemEval Semantic Textual Similarity (SemEval STS) challenge has been organized for over five years, which calls for effective models to measure sentence similarity [20]. Averaged word embeddings are used as a baseline to measure sentence pair similarity in the challenges: each sentence is transformed into a vector by averaging the word vectors for each word in the sentence, and sentence pair similarity is effectively measured by the similarity between the averaged vectors using common measures such as Cosine and Euclidean similarity.

Instead of training the word vectors from scratch, we leverage the pre-trained biomedical word embeddings (BioWordVec-intrinsic) that are trained on a PubMed text corpus and MeSH data [50]. The rationale is to “precondition” the Siamese network with prior knowledge of the inherent similarity between words in the UMLS vocabulary. Prior to generating the positive and negative pairs, we preprocess the lexical features of UMLS atoms similar to how the authors in [50] preprocessed their dataset (i.e., we removed all punctuation except hyphen, lowercased, and tokenized on space) to ensure conformity as we leverage their pre-trained BioWordVec embeddings in our downstream network.

Upon plotting a word length distribution, 97% of atoms in the UMLS have a word length of 30 or less. Hence, we apply padding or truncation to restrict the word length of each atom to a maximum of 30 to ensure a uniformity in dimension to speed up the training process. The embeddings of the pair of atoms are fed to two *LSTMs*, each of which processing one of the atoms in the pair and consisting of 50 hidden learning units. These units learn the specific semantic and syntactic features based on word order of each individual atom through time.

#### Siamese-LSTM network.

Contrary to the traditional neural networks which accepts one input at a time, the Siamese network is an architecture that takes a pair of inputs and learns representations based on explicit similarity and dissimilarity information (i.e., the pairs of similar and dissimilar inputs) [5]. It was originally used for signature verification [5] and has since been applied to various applications such as face verification [6], unsupervised acoustic modeling [43], and learning semantic entailment [32], as well as text similarity [34].

A series of deep learning (DL) models can be incorporated within the Siamese architecture. RNNs (Recurrent Neural Networks) are a type of DL model that excel at processing sequential information due to the presence of memory cells to store and “remember” data read over time [40]. A particular variant of RNN is the Long Short-Term Memory (LSTM). It enhances the standard RNN to handle long-term dependencies and to minimize the inherent vanishing gradient problem of RNNs with the introduction of “gates” (input, output, and forget gates) to control the flow of and retain information better through time. It is more accurate in handling long sequences. However, it comes at the cost of higher memory consumption and longer training times compared to a standard RNN which is faster, but less accurate. Nonetheless, a combination of a Siamese network with RNN and LSTM have been successfully applied to various NLP tasks including similarity assessment [12, 32, 44]. On the other hand, CNNs (Convolutional Neural Networks) have also performed well in NLP due to their ability to extract distinctive features at a higher granularity [20]. A Siamese CNN model learns sentence embedding and predicts sentence similarity with features from various convolution and pooling operations [15].

The output of the model is a Manhattan distance similarity function, *exp*(−‖*LSTM_A_* − *LSTM_B_* ‖_1_) ∈ [0, 1], a function that is well-suited for high dimensional spaces [1]. We will use the Siamese neural network architecture with LSTM and the datasets described above to train our models. Next, we describe our design for evaluating the supervised learning approach and comparing it with the rule-based approach.

## 5 EVALUATION

This section presents the experiments to evaluate the proposed supervised learning approach against the baseline from the rule-based approach.

The experiments are reproducible and the baselines are also reusable. The materials for reproducing the experiments are publicly available. A no-cost UMLS license^1^ is required to access and download the materials in this page.

### 5.1 Experimental Setup

We conducted two types of experiments on the same datasets and evaluated the performance of (1) the rule-based approximation baseline, and (2) the proposed supervised learning approach. The editorial rules are defined Section 3.2 and the neural networks are described in Section 4. We implemented our approaches using Python 3.8 and Tensorflow 2.0.

Both experiment types are executed by deploying batches of parallel jobs to the Biowulf high-performance computing cluster^2^ at the National Institutes of Health (NIH). We use the *norm* and *gpu* partitions for the corresponding CPU and GPU servers in this cluster with a limit of 10,000 CPU cores, 60 TB of RAM, and 56 GPUs per user. Our evaluation includes several steps organized into different pipelines. The execution of each step maximizes the resources allocated in Biowulf to reduce the runtime. Our settings for deployment are: (1) using multiple nodes, usually 500-625 nodes, (2) using multiple threadings with 16-20 threads per node, (3) using about 95-125 GB of RAM per node, and (4) using Tesla V100 GPUs for the training and testing tasks.

The implementation is highly configurable, reusable, and reproducible with scripts. However, note that these experiments make extensive use of computational resources. We reportedly used over 1.6 million CPU hours over 3 months for developing and deploying the models.

### 5.2 Data Generation

We used the active source vocabularies restricted to English terms (excluding suppressible synonyms) in the UMLS 2020AA release, which can be downloaded ^3^ with a no-cost UMLS license^1^.

Table 3 shows the statistics for the 8 datasets generated for the experiments with 4 dataset variants (*TOPN_SIM, RAN_SIM, RAN_NOSIM, ALL*). Each variant has one dataset for learning the models and one dataset for testing the generalization of the models as described in Section 4.2. The process of generating these datasets, especially for the *TOPN_SIM* and *RAN_SIM* variants, involves the computation of lexical similarity scores for all pairs in the Metathesaurus, i.e, 7.6e+13 pairs. This number of pairs is extremely intensive to compute. We took advantage of the normalized word index in the Metathesaurus for reducing the workload. This index links each AUI to normalized words that form the basis for our similarity computation. Therefore, for every AUI, we only need to compute the similarity scores against a small fraction of all of the other AUIs (8.7M) sharing at least one normalized word and select a number of pairs with top scores for *TOPN_SIM* and *RAN_SIM.* This operation required approximately 10,000 CPU cores in the Biowulf cluster, but finished within 20 hours.

### 5.3 Rule-based Approximation Baseline

We implemented the editorial rules defined in Section 3.2. For evaluating how individual and combined rules influence the performance, we created four variants of the RBA baseline: (1) *SS* for the source synonymy rule, (2) *LS_SC* for the lexical similarity and semantic compatibility rule, (3) *SS_LS_SC* for the disjunction of the two *SS* and *LS_SC* rules (*SS* OR *LS_SC*), and (4) *SS_LS_SC_TRANS* for the transitive closure of the *SS_LS_SC* variant. We evaluate and compare the four RBA variants using the 4 variants of the generalization dataset. We will select the best RBA variant as our baseline for comparison against the supervised learning approach.

#### Results.

Table 4 shows the results of the evaluation. All the RBA variants consistently share the same pattern across all the generalization datasets, namely very high precision (0.8631 to 1), but very low recall (0.2026 to 0.6871). Comparing the performance of these RBA variants against the 4 variants of the generalization dataset, each RBA variant shares the same recall for all the generalization datasets, while precision and F1 score improve among *ALL, TOPN_SIM, RAN_SIM*, and *RAN_NOSIM*.

The *SS_LS_SC_TRANS* variant performed best in terms of accuracy, recall, and F1 score, but had the lowest precision among all the RBA variants across all the generalization datasets. Adding the transitive closure (*SS_LS_SC_TRANS* variant) significantly increased the performance with a 16% increase in recall and 19-23% in F1 score across all the generalization datasets. The *SS* rule yields higher precision and recall compared to the *LS_SC* rule. Combining the two rules with OR (*SS_LS_SC* variant) also brings significant improvements with a 18% increase in recall and 19% in F1 score. We will compare this SS_LS_SC_TRANS variant with the deep learning approach in Section 5.5.

### 5.4 Training

#### Training parameters.

For training the neural networks, we ran various experiments to select the most suitable hyper-parameters that can balance performance and speed for our models. We tried batch sizes from 64 to 65356 and learning rates from 0.00001 to 0.01. While a batch size of 64 can take at least 16 hours of training for an epoch with a single V100 GPU, a batch size of 8192 can finish an epoch in less than 10 minutes. Also, the experiments in [49] suggest to fit as many data samples as possible to the GPU memory, but not higher than 8192. This was consistent with our preliminary findings. Therefore, we used a batch size of 8192 in our experiments.

#### Trained Model Variants.

We split each variant (*ALL, TOPN_SIM, RAN_SIM*, and *RAN_NOSIM*) of the dataset into a training dataset (75%) and a validation dataset (25%). We trained and evaluated each variant with 100 epochs and report the results in Table 5 with the usual metrics (accuracy, precision, recall, and F1 score).

#### Results.

As shown in Table 5, all the trained models can learn very effectively. Accuracy, precision, recall, and F1 score exceed 93% for training and validation. We observed that compared to other models, the *TRAINED_RAN_NOSIM* model was able to learn especially well with all the metrics near or above 99% and low loss. This was expected because its input pairs are highly dissimilar lexically and mostly non-synonymous. Training seems less effective when the negative input pairs were more lexically similar but non-synonymous, like the ones in *TRAINED_ALL* and *TRAINED_TOPN_SIM*. Of note, the excellent training scores from the *TRAINED_RAN_NOSIM* do not guarantee good generalization, as we show in the next section.

### 5.5 Generalization Test Results

This section provides a comprehensive performance comparison between the trained models (*TRAINED_ALL, TRAINED_TOPN_SIM, TRAINED_RAN_SIM*, and *TRAINED_RAN_NOSIM*), and the rule-based approximation baseline (*SS_LS_SC_TRANS*) using the same generalization datasets. Since each model is trained with a dataset corresponding to a specific variant in terms of lexical similarity between atoms in the negative pairs, we perform a generalization test by evaluating the model performance on generalization datasets for other variants of lexical similarity in negative pairs. Table 6 shows the results of the performance comparison. Here we compare the trained models with each other and against the rule-based approximation *SS_LS_SC_TRANS*.

#### Comparing DL-trained models.

As shown in Table 6, the *TRAINED_RAN_NOSIM* variant seemed to perform very well with its own generalization variant *RAN_NOSIM* with all of the metric scores being above 97.9%. However, it did not generalize well to other test variants, especially the *ALL* and *TOPN_SIM*, with very low precision 20-22%. The *TRAINED_RAN_SIM* model had a performance pattern similar to the *TRAINED_RAN_NOSIM* model, but with 20-23% improvement in F1 score for the *ALL* and *TOPN_SIM* generalization variants.

In contrast, compared to the two *RAN* models above, the two models *TRAINED_ALL* and *TRAINED_TOPN_SIM* had exceptionally good performance in every measure across all the generalization variants. Of the two, the *TRAINED_ALL* model had consistently better results than the *TRAINED_TOPN_SIM* in every measure. Overall, the performance for the trained models ranked as follows from worst to best: *TRAINED_RAN_NOSIM, TRAINED_RAN_SIM, TRAINED_TOPN_SIM*, and *TRAINED_ALL.*

These experiments show that the degrees of lexical similarity (*ALL, TOPN_SIM, RAN_SIM, RAN_NOSIM*) between strings in negative pairs actually influence performance, thus confirming our hypothesis. Learning from one of the lexical similarity variants is necessary, but insufficient. The trained models without *TOPN_SIM* pairs perform worse than the trained models with those pairs, which demonstrates the importance of the highest lexical similarity variant. The *TRAINED_TOPN_SIM* model without *RAN_SIM* and *RAN_NOSIM* pairs perform worse than the *TRAINED_ALL* model with those pairs, which demonstrates the importance of the *RAN_SIM* and *RAN_NOSIM* pairs. The *TRAINED_ALL* model combining all three degrees yields the best performance. Next, we will compare the *TRAINED_ALL* model with the best RBA variant.

#### Comparing the best trained model *TRAINED_ALL* with the best RBA variant *SS_LS_SC_TRANS*.

Overall, the *TRAINED_ALL* model consistently outperforms the rule-based *SS_LS_SC_TRANS* variant by a large margin in every measure. The best RBA variant has high precision and low recall, while the best DL-trained model has both high precision and high recall across all the generalization variants. While their accuracy and precision are quite close (1-3%), there are significant differences in their recall (21-22%) and F1 score (23-24% for *ALL* and *TOPN_SIM*, 11-14% for *RAN_SIM* and *RAN_NOSIM*).

#### Comparing prediction differences.

Here we analyze those cases where the DL and RBA approaches make different predictions in the *ALL* generalization dataset. Table 7 shows the distribution of correct and incorrect predictions in the *SIM* and *NOSIM* sets. Overall, while the RBA approach makes a larger number of wrong predictions than the DL approach, both approaches tend to have more difficulty making accurate predictions for pairs with a some lexical similarity (*SIM*) compared to pairs with no lexical similarity (*NOSIM*). This is consistent with our assumption that highly similar but non-synonymous pairs are more difficult to predict.

### 5.6 Overall Discussion

#### Findings.

The experimental evaluation presented above has shown that a relatively simple DL approach largely outperformed the best variant of the rule-based approximation approach. It has also validated our hypothesis that lexical similarity degrees among negative pairs strongly influence the performance of the trained models. However, the DL approach did take longer time for prediction than the RBA approach. Particularly, the DL models took about an hour for predicting the generalization test sets with a single V100 GPU while the best RBA variant took 15-20 minutes with a CPU server.

#### Significance.

Compared to the rule-based approximation, the excellent performance of the *TRAINED_ALL* model is even more remarkable given that it only uses lexical information (e.g., terms) from the source vocabularies, while the rule-based approach uses both lexical information and contextual information (i.e., source synonymy and semantic group). These results suggest that the DL approach could be further improved by incorporating contextual information. Furthermore, the good performance of the DL approach on pairs with no lexical similarity (above 95% for F1 and 99% for accuracy) encourages us to perform more extensive experiments on the UMLS, where most pairs exhibit no lexical similarity.

#### Limitations and Future Work.

There are several limitations to this preliminary investigation, which we plan to address in future work. As mentioned earlier we have not yet incorporated contextual information into the neural networks, which we could do by using additional vectors for the terms of neighboring concepts or by using Graph Neural Networks for representing relations among atoms, such as source synonymy and hierarchical relations. Also, we have not yet evaluated the approaches at the full-scale of the UMLS Metathesaurus. While a full-scale evaluation is extremely expensive computationally (10^14^ pairs), we plan to perform larger evaluations in the future. We also need to perform an error analysis to better understand how learning could be improved. Finally, we deliberately used fairly simple and established DL techniques in this work. In the future, we plan to experiment with recent techniques, such as transformers (e.g., BioBERT), which we briefly discuss in the next section.

#### Generalization.

Beyond the confines of the UMLS project, our approach can be used in a variety of terminology integration and ontology alignment applications in biomedicine and healthcare. For example, BioPortal [37] is “the world’s most comprehensive repository of biomedical ontologies”. It uses lexical similarity to find equivalent terms among ontologies. It would be interesting to test our DL approach on this vast repository. Along the same lines, we plan to test our approach on biomedical ontologies in the ontology alignment evaluation organized by OAEI. We also expect that other researchers will be encouraged to try similar approaches for ontology alignment outside the biomedical domain, provided sufficient material is available for learning purposes.

#### Applications.

This research is directly applicable to improve the UMLS construction process. Two applications come to mind, which we will be exploring shortly. The first one is the insertion of new source vocabularies (or new terms from updated source vocabularies) into the Metathesaurus as part of the bi-annual Metathesaurus update process. Predictions from our DL approach could replace the rule-based predictions and be presented to human editors, hopefully saving them time compared to the current editing environment. Another, more ambitious application is to “rebuild the Metathesaurus from scratch”. What we envision is to use our pairwise synonymy prediction to cluster atoms in a manner to recreate the Metathesaurus concepts. The analysis of differences with the existing Metathesaurus could open interesting avenues for quality assurance.

## 6 RELATED WORK

The OAEI has been driving ontology matching research in the biomedical domain since 2005. The *largebio* track uses the datasets extracted from a subset of source vocabularies in the UMLS Metathesaurus. A variety of matching techniques including rule-based and statistical methods have been developed. Among the top general-purpose matchers are AgreementMakerLight (AML) [10], YAM++ [35], and LogMap [17]. AML [10] uses a combination of different matchers, such as the lexical matcher, mediating matcher, and word-based string similarity matcher. YAM++ [35] implemented a decision tree learning model over many string similarity metrics but leaves the challenges of finding suitable training data to the user, defaulting to information retrieval-based similarity metrics for its decision-making when no training data is provided. LogMap [17] is designed to efficiently align large ontologies, generating logical output alignments.

Similarity assessment between words and sentences, also known as Semantic Text Similarity (STS) task, is an active research area in Natural Language Processing (NLP) due to its crucial role in various downstream tasks such as information retrieval, machine translation, and in our case, synonym clustering. The STS task can be expressed as follows: given two sentences, a system returns a probability score of 0 to 1 indicating their degree of similarity. STS is a challenging task due to the inherent complexity in language expressions, word ambiguity, and variable sentence lengths. Traditional approaches rely on hand-engineering lexical features (e.g., word overlap and subwords [22], syntactic relationship [51], structural representations [42]), linguistic resources (e.g., corpora), bag-of-words and term frequency-inverse document frequency (TF-IDF) models that incorporate a variety of similarity measures [11] for example string-based [13] and term-based [41]. However, most are syntactically and semantically constrained.

Recent successes in STS [29] in predicting sentence similarity and relatedness have been obtained by using corpus-based [23] and knowledge-based similarity, e.g. word embeddings for feature representation [27] with supervised DL approaches, e.g., Siamese Network with Recurrent Neural Network (RNN) [32] and Convolutional Neural Networks (CNN) [15] as well as hybrid approaches [16] to perform deep analysis of words and sentences to learn the necessary semantics and structure. Unsupervised attention and transformer-based mechanisms that were pioneered by Google research [46] have also been widely applied to STS with great degree of success [38]. The (self)-attention mechanism adds attention, weights keywords, learns contextual relations between words (or sub-words) in a text, and finds the connection within the sequence of words [14]. One of such transformer-based computations is Bidirectional Encoder Representations (BERT) which has consistently triumphed in most NLP tasks including STS [9]. Other variants trained on different corpora include BioBERT, which was pre-trained on the PubMed text corpus, has outperformed many biomedical-related NLP tasks [24]. This form of two-step-learning (pre-training and fine-tuning), termed transfer learning, is a popular method where a model trained on general domain with large-scale well-annotated datasets is re-purposed as the starting point for a model on a second (related) task. In our DL approach, we employed this form of learning by using pre-trained biomedical word embeddings (from BioWordVec-intrinsic) and subsequently fine-tuned the network with Bi-LSTM(s). Since this is the first contribution (to the best of our knowledge) in applying DL to biomedical vocabulary alignment task at scale, we adopted a knowledge-based similarity approach (Siamese-BioWordVec-BiLSTM network) for its simplicity and effectiveness. We aimed to evaluate this approach on real-world data and against a rule-based approximation of the current Metathesaurus construction process, instead of benchmarking it against other forms of resource-intensive DL techniques, such as attention and transformer-based mechanisms in the future work.

Reminiscent of the UMLS are two projects that aim to discover and organize links among large knowledge resources, BabelNet [33] and LIMES [36]. Closest to our work is a recently published paper in which the authors used DL techniques to measure semantic relatedness in the UMLS Metathesaurus [25]. There are, however, several major differences with our work, including the fact that they assessed semantic relatedness among concepts, while we assess synonymy among atoms. In addition, the scale of their work is limited to a few thousands of UMLS concept pairs, while the number of atom pairs involved in our experiments is several orders of magnitude larger.

## 7 CONCLUSION

We have presented our supervised approach for learning synonymy between biomedical terms in the UMLS Metathesaurus. The excellent performance of the supervised learning model compared to the rule-based approximation of the UMLS Metathesaurus construction process used as our baseline shows the great potential of this learning approach, especially because the learning approach only makes use of the lexical features (terms) from the source vocabularies, while the rule-based approach additionally uses contextual information (source synonymy and semantics). This approach has great potential for improving the UMLS Metathesaurus construction process by providing better synonymy suggestions to the UMLS editor.

## Figures and Tables

**Table 1:** Examples of atoms from a Metathesaurus concept, with associated identifiers and semantic group

Tuple	String	Source	SCUI	AUI	SUI	LUI	CUI	Semantic Group
*t*_1_	Headache	MSH	M0009824	A0066000	S0046854	L0018681	C0018681	Disorders
*t*_2_	Headaches	MSH	M0009824	A0066008	S0046855	L0018681	C0018681	Disorders
*t*_3_	Cranial Pains	MSH	M0009824	A1641924	S1680379	L1406212	C0018681	Disorders
*t*_4_	Cephalodynia	MSH	M0009824	A26628141	S0475647	L0380797	C0018681	Disorders
*t*_5_	Cephalodynia	SNOMEDCT_US	25064002	A2957278	S0475647	L0380797	C0018681	Disorders
*t*_6_	Headache (finding)	SNOMEDCT_US	25064002	A3487586	S3345735	L3063036	C0018681	Disorders

**Table 2:** List of abbreviations used in the paper

Notion	Meaning	Notion	Meaning
*SRC*	Vocabulary source	*T*	set of tuples
*STR*	Atom string	*S_SRC_*	set of *SRCs*
*AUI*	Atom unique ID	*S_STR_*	set of *STRs*
*CUI*	Concept unique ID	*S_AUI_*	set of *AUIs*
*LUI*	Lexical unique ID	*m_a_*	*S_STR_* x *S_SRC_* → *S_AUI_*
*SUI*	String unique ID	*S_SUI_*	set of SUIs
*SCUI*	Source *CUI*	*m_s_*	*S_AUI_* → *S_SUI_*
*SS*	Source synonym	*S_LUI_*	set of *LUIs*
*LS*	Lexical similarity	*m_l_*	*S_SUI_* → *S_LUI_*
*SC*	Semantic compatibility	*S_SCUI_*	set of SCUIs
*SG*	Semantic group	*m_u_*	*S_AUI_* → *S_SCUI_*
*TRANS*	Transitivity	*S_SG_*	set of *SGs*
*SIM*	Similarity	*m_g_*	*S_AUI_* → *S_SG_*
*NOSIM*	No similarity	*m_c_*	*S_AUI_* → *S_CUI_*
*RAN*	Random		

**Table 3:** Dataset statistics from different similarity variants (JACC = 0: no similarity, JACC > 0: some similarity)

		LEARNING			GENERALIZATION		
PER AUI	VARIANT	NEG	POS	TOTAL	NEG	POS	TOTAL
pairs with top JACC scores	TOP_SIM	55,909,551	22,324,834	78,234,385	54,752,228	5,581,209	60,333,437
random pairs with JACC > 0	RAN_SIM	55,909,551	22,324,834	78,234,385	54,445,899	5,581,209	60,027,108
random pairs with JACC = 0	RAN_NOSIM	58,256,526	22,324,834	80,581,360	58,256,526	5,581,209	63,837,735
include all the above	ALL	170,075,628	22,324,834	192,400,462	167,454,653	5,581,209	173,035,862

**Table 4:** Performance results for the four variants of the Rule-based Approximation baseline applied to each of the four variants of the generalization dataset (SS: Source Synonym, LS: Lexical Similarity, SC: Semantic Compatibility)

	Accuracy				Best F1			
	Generalization Variant				Generalization Variant			
RBA Variant	ALL	TOPN_SIM	RAN_SIM	RAN_NOSIM	ALL	TOPN_SIM	RAN_SIM	RAN_NOSIM
SS_LS_SC_TRANS	**0.9863**	**0.9614**	**0.9702**	**0.9726**	**0.7651**	**0.7672**	**0.8109**	**0.8145**
SS_LS_SC	0.9806	0.9443	0.9453	0.9486	0.5776	0.5777	0.5833	0.5834
SS	0.9752	0.9290	0.9290	0.9332	0.3808	0.3808	0.3818	0.3819
LS_SC	0.9739	0.9252	0.9258	0.9303	0.3339	0.3340	0.3369	0.3369
	Precision				Recall			
	Generalization Variant				Generalization Variant			
RBA Variant	ALL	TOPN_SIM	RAN_SIM	RAN_NOSIM	ALL	TOPN_SIM	RAN_SIM	RAN_NOSIM
SS_LS_SC_TRANS	0.8631	0.8683	0.9892	0.9999	**0.6871**	**0.6871**	**0.6871**	**0.6871**
SS_LS_SC	0.9663	0.9669	0.9993	1	0.4119	0.4119	0.4119	0.4119
SS	**0.9854**	**0.9857**	**0.9997**	**1**	0.2360	0.2360	0.2360	0.2360
LS_SC	0.9491	0.9500	0.9990	1	0.2026	0.2026	0.2026	0.2026

**Table 5:** Training and validating (V_) results for 4 trained model variants from the learning datasets at epoch = 100 and batch size = 8192

DL Model Variant	LOSS	V_LOSS	ACC	V_ACC	PRE	V_PRE	RECALL	V_RECALL	F1	V_F1
TRAINED_ALL	0.0159	0.0199	0.9852	0.9799	0.9464	0.9318	0.9496	0.9256	0.9480	0.9287
TRAINED_TOP_SIM	0.0339	0.0427	0.9678	0.9553	0.9448	0.9285	0.9595	0.9381	0.9521	0.9333
TRAINED_RAN_SIM	0.0095	0.0147	0.9925	0.9857	0.9880	0.9819	0.9894	0.9749	0.9887	0.9784
TRAINED_RAN_NOSIM	**0.0035**	**0.0064**	**0.9973**	**0.9935**	**0.9982**	**0.9956**	**0.9935**	**0.9843**	**0.9958**	**0.9899**

**Table 6:** Comparing the performance between 4 DL training model variants and the best RBA variant (SS_LS_SC_TRANS) across all the generalization datasets

	Accuracy				Best F1			
	Generalization Variant				Generalization Variant			
DL Model variant and RBA	ALL	TOPN_SIM	RAN_SIM	RAN_NOSIM	ALL	TOPN_SIM	RAN_SIM	RAN_NOSIM
TRAINED_ALL	**0.9938**	**0.9807**	0.9905	0.9924	**0.9061**	**0.8974**	0.9469	0.9549
TRAINED_TOP_SIM	0.9844	0.9750	0.9831	0.9861	0.7954	0.8740	0.9117	0.9217
TRAINED_RAN_SIM	0.9497	0.8627	**0.9935**	0.9960	0.5572	0.5678	**0.9654**	0.9768
TRAINED_RAN_NOSIM	0.8695	0.6753	0.9526	**0.9968**	0.3286	0.3593	0.7943	**0.9816**
SS_LS_SC_TRANS	0.9863	0.9614	0.9702	0.9726	0.7651	0.7672	0.8109	0.8145
	Precision				Recall			
	Generalization Variant				Generalization Variant			
DL Model variant and RBA	ALL	TOPN_SIM	RAN_SIM	RAN_NOSIM	ALL	TOPN_SIM	RAN_SIM	RAN_NOSIM
TRAINED_ALL	**0.8875**	**0.8841**	0.9858	0.9971	0.9254	0.9110	0.9110	0.9162
TRAINED_TOP_SIM	0.6908	0.8182	0.8868	0.9059	0.9375	0.9380	0.9380	0.9380
TRAINED_RAN_SIM	0.3901	0.4005	0.9560	0.9787	0.9748	0.9750	0.9750	0.9750
TRAINED_RAN_NOSIM	0.1972	0.2197	0.6658	0.9790	**0.9843**	**0.9842**	**0.9842**	**0.9842**
SS_LS_SC_TRANS	0.8631	0.8683	**0.9892**	**0.9999**	0.6871	0.6871	0.6871	0.6871

**Table 7:** Comparing prediction differences from the best variants of Deep Learning models (*TRAINED_ALL*) and Rule-based Approximation baseline (*SS_LS_SC_TRANS*) on the same *ALL* generalization dataset

Label	DL prediction	RBA prediction	Similarity	Number of pairs
1	1	0	NOSIM	187,843
1	1	0	SIM	1,439,307
0	0	1	NOSIM	15,111
0	0	1	SIM	524,724
1	0	1	NOSIM	97,876
1	0	1	SIM	196,922
0	1	0	NOSIM	744
0	1	0	SIM	496,804

## Footnotes

1
https://uts.nlm.nih.gov/home.html

2
https://hpc.nih.gov/

3
https://www.nlm.nih.gov/research/umls/licensedcontent/umlsknowledgesources.html
